# Janus flame-retardant polyester fabric with one-way water transfer for fog collection: Influence of plasma duration, aluminum phosphate, and TiO_2_ nanoparticles

**DOI:** 10.1016/j.heliyon.2025.e42400

**Published:** 2025-01-31

**Authors:** Najmeh Izadi, Majid Montazer, Aminoddin Haji

**Affiliations:** a Textile Engineering Department, Functional Fibrous Structures & Environmental Enhancement (FFSEE), Amirkabir University of Technology (AUT), Tehran, Iran; b Department of Textile Engineering, Yazd University, Yazd, Iran

**Keywords:** Janus polyester fabric, Fog collection, Flame retardant, Plasma treatment, Aluminum phosphate

## Abstract

Significant efforts have been made to create Janus fabrics including designing their structure, employing electrospraying and electrospinning, and applying chemical and physical surface treatments to enhance the effective and directed transport of water. In this paper, a Janus industrial fabric with desirable properties such as one-way water transfer, fog collection, mechanical durability, and flame resistance was prepared. The creation of superhydrophobic surfaces on polyester fabrics involved the attachment of titanium dioxide nanoparticles (NTO) onto the fabric using aluminum phosphate (AP) adhesive and then coating with fluoroacrylate polymer (F) as a low-energy surface material. One-sided O_2_/Ar plasma technology was used on a superhydrophobic fabric to generate asymmetric wettability. As plasma duration rises, the depth of surface modification and oxidation intensifies. By altering the duration of plasma exposure, this fabric gained the ability to trap, transport, and collect fog. At an exposure duration of 3 min, the fabric attained one-way water transfer. The water contact angle (WCA) on the hydrophilic side was 38°, whereas the WCA on the opposing side was 120°. The fog collecting efficiency at this time was approximately 8.1 mg cm^−2^ min^−1^. FESEM and EDX confirmed the distribution and adhesion of NTO to the fabric surface with AP. AP gave the fabric great flame resistance and a limiting oxygen index (LOI) of 32.8 %. Mechanical properties were unaffected, but air permeability and the CIE whiteness index decreased.

## Highlights

• Janus fabric through a simple method using NTO, AP, fluoropolymer, and plasma treatment.
• Optimization of plasma exposure time for one-way water transfer of water-repellent fabric.
• Exceptional flame-retardant characteristics on the Janus polyester fabric.
• No significant changes in the mechanical properties of the fabric.

## Graphical abstract

**Figure:**
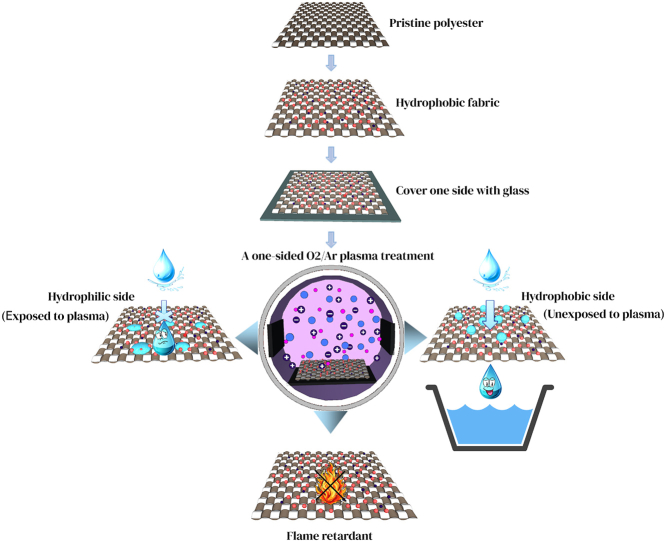

**Table:** Abbreviations

NTO	Titanium Dioxide Nanoparticles
AP	Aluminum Phosphate
F	Fluoroacrylate polymer
WCA	Water Contact Angle
LOI	Limiting Oxygen Index
O_2_/Ar	Oxygen/Argon
HMDSO	Hexamethyldisiloxane
PDMS	Polydimethylsiloxane
Cu	Copper
MMT	Moisture Management Tester
FESEM	Field Emission Scanning Electron Microscope
EDX	Energy Dispersive X-ray
η	Fog collection efficiency
M	Mass of water collection
A	Surface area of the sample
t	Fog collection time
R	One-way transport capacity
C	Carbon
O	Oxygen
Al	Aluminum
P	Phosphorus
Ti	Titanium
ΔP	Intrusion pressure
γ	Interfacial tension
θ	Advancing water contact angle
R	Radius of pore
HF	Hydrophobic Force
HP	Hydrostatic Pressure
CF	Capillary Force
AP-F@3	Treated polyester fabric with aluminum phosphate and fluoroacrylate polymer, then exposed to plasma for 3 min
AP-NTO-F@3	Treated polyester fabric with aluminum phosphate, titanium dioxide nanoparticles, and fluoroacrylate, polymer then exposed to plasma for 3 min
F@3	Treated polyester fabric with fluoroacrylate polymer then exposed to plasma for 3 min.

## 1 Introduction

In recent years, researchers have shown significant interest in Janus fabrics. The term "Janus" originates from the ancient Roman concept of deities with dual faces, positioned in opposite directions. This symbolism signifies their ability to both observe the past and predict the future [1]. These materials exhibit different characteristics from two sides, including heating-cooling, hydrophilic-hydrophobic, and combining different functions on the same object [2,3]. Janus fabrics have asymmetric wettability, enabling the one-way transfer of water. This allows the movement of water from the hydrophobic side to the hydrophilic side while preventing the transfer of water to the inverse side [4]. Significant efforts have been dedicated to creating Janus fabrics, which involve designing their structure [5], employing electrospraying [6] and electrospinning [7], and applying chemical [8] and physical [9] surface treatments to enhance the effective and directed transport of water.

Plasma treatment is a physical finishing method used to alter the surface properties of materials while minimizing any damage to their bulk properties. Plasma is rich in reactive species, including negative and positive ions, ultraviolet photons, excited atoms and molecules, free radicals, and electrons. These species have different effects on fabric, including corrosion and a rough surface, the generation of free radicals, and the alteration of hydrophilic and hydrophobic functional groups [10,11]. The characteristics of plasma-treated surfaces are dictated by the precursor gases derived from polymeric or non-polymeric gases, as well as the reaction parameters such as flow rate, time, pressure, power, and frequency [12]. Po et al. prepared Janus fabrics by plasma-depositing hexamethyldisiloxane (HMDSO) onto one side of a cotton nonwoven substrate. HMDSO was polymerized using the energy produced by the plasma and subsequently deposited onto the surface. By controlling the plasma process, the treated surface became hydrophobic, while the untreated surface remained hydrophilic [13]. Here, the plasma treatment time was studied to create hydrophilicity on one side of the polyester fabric, which has poor adhesion and a low moisture sorption rate. Fabrics with directional transfer water properties can be used to collect fog. This fog-collection design offers a highly effective solution for prevailing potential water scarcity in the future [14]. It offers an energy-efficient approach to control the movement of liquids without external energy sources, pumps, and condensers [15]. Malik et al. reviewed those plants and animals directly absorbing water from the atmosphere [16]. Also, researchers have developed water collectors that mimic biological systems [17]. The water collection method contains three portions: “water trap, water collection, and water transfer” [18]. These collectors include fog collectors with hydrophobic-hydrophilic patterns, water-collecting filaments, and Janus textiles with contrast in wettability in thickness [[19], [20], [21]]. Zhu et al. fabricated fabrics with alternating hydrophobic and hydrophilic patterns by utilizing the photocatalytic degradation capabilities of PDVB under UV irradiation using lithographic masks with different holes. The resulting fabrics exhibited a fog harvesting rate of 224.7 mg cm^−2^ h^−1^ [22].

Flame resistance is an extra characteristic that enhances the application of a coating in more severe conditions. Flame retardant finishing is a very efficient and simple technique for applying polyester fabric [23]. To achieve this feature, various compounds are utilized, including halogenated compounds, particularly those containing bromine. Nevertheless, they were unable to improve the resistance to dripping, and their utilization is prohibited due to environmental concerns. Instead, compounds containing phosphorus are employed [24]. AP is appropriate for producing flame-retardant coatings because of its exceptional resistance to high temperatures and its ability to adhere firmly [25]. Xue et al. the cotton fabrics were modified to have superhydrophobic surfaces by attaching polystyrene nanoparticles using AP, then by applying a coating of polydimethylsiloxane (PDMS) as a substance with low surface energy. These surfaces exhibited high mechanical durability. However, their investigation did not include an examination of the flame-retardant properties of these fabrics [26]. The application method and results of some reports on Janus substrates are summarized in Table 1. The application of organic and inorganic adhesives to attach NTO to polyester fabric, combined with one-side plasma treatment to establish a wettability gradient, introduces an innovative approach for producing flame-retardant polyester Janus fabric. Further, the influence of plasma duration on the continuous one-way water flow of this fabric may serve as a simple and cost-effective method to enhance water transfer.

**Table 1:** Fabrication and properties of Janus substrates.

Substrate	Method	Function and application	Ref.
PET	Introduced superhydrophilic Cu(OH)₂ nanowires on PET	One-way water transfer, water-collecting efficiency of 467.3 mg cm^−2^ h^−1^	[27]
Polyester fabric	Superhydrophobic coating and UV irradiation	Unidirectional water-transfer	[28]
Polyester fabric	Electrospraying	Directional oil transport	[29]
Polyester filter nets	Sandwiching a hydrophilic layer between two hydrophobic layers	Fog-collecting efficiency of 0.33 g cm^−2^h^−1^	[30]
Stainless steel meshes	Applying NTO suspension combined with AP adhesive and UV irradiation	Underwater superoleophobic surfaces, oil/water separation, and self-cleaning properties	[31]
Ramie	Chitosan and phytic acid deposition followed by polydivinylbenzene coating and one side UV irradiation	Flame-retardant, unidirectional liquid movement, moisture-winking, and oil/water separation	[32]
Cotton fabric	Super-hydrophobic coating and plasma treatment	Dermal-like textile with one-way water transfer	[33]

Mono-functional industrial fabrics only have one specific application, which limits their usefulness [34]. Additionally, there is a lack of research on how to create industrial fabrics with several functions. Hence, the advancement of multifunctional textiles has assumed great significance in the textile industry. This study generated a multifunctional textile by adhering NTO to the surface of polyester fabric using an economical mineral adhesive, AP, followed by one-sided plasma technology. The influence of O_2_/Ar plasma duration on the one-way water transport characteristics was also examined. With an increase in plasma duration, the depth of surface modification and oxidation escalated, altering the hydrophilicity level of the surface and consequently influencing the water transport behavior of the fabric. This Janus industrial fabric possesses multiple desirable features, such as unidirectional water transfer, fog collecting, mechanical stability, and flame retardant. Also, employing AP provided the fabric with flame-retardant properties and prevented dripping. Thus, this innovative method paves the way for the creation and development of textiles with multifunctional properties. These textiles have considerable potential for diverse applications such as functional covers and the production of fresh water from water vapor.

## 2 Experimental

### 2.1 Materials

Polyester fabric with a plain texture of 94 g m^−2^ and a thickness of 240 μm was provided by Yazd Baft Co., Yazd. Phosphoric acid (H_3_PO_4_) and aluminum hydroxide (Al(OH)_3_) were purchased from Merck Co., Germany. The fluoropolyacrylate compound, known as Bit-guard FC, was acquired from Supross Co., Switzerland. Nano titanium dioxide (Degussa P-25, 21 nm) was applied from Evonik Co., Germany.

### 2.2 Preparation of polyester fabric

The fabric was washed using a solution containing 1 g L-1 of nonionic detergent. It was carried out at 60 °C for 20 min to eliminate spinning oils, waxes, and soluble impurities. The fabric was then washed with distilled water and allowed to air dry at room temperature.

### 2.3 Fabrication of a superhydrophobic coating on polyester fabric

**Fig. 1:**
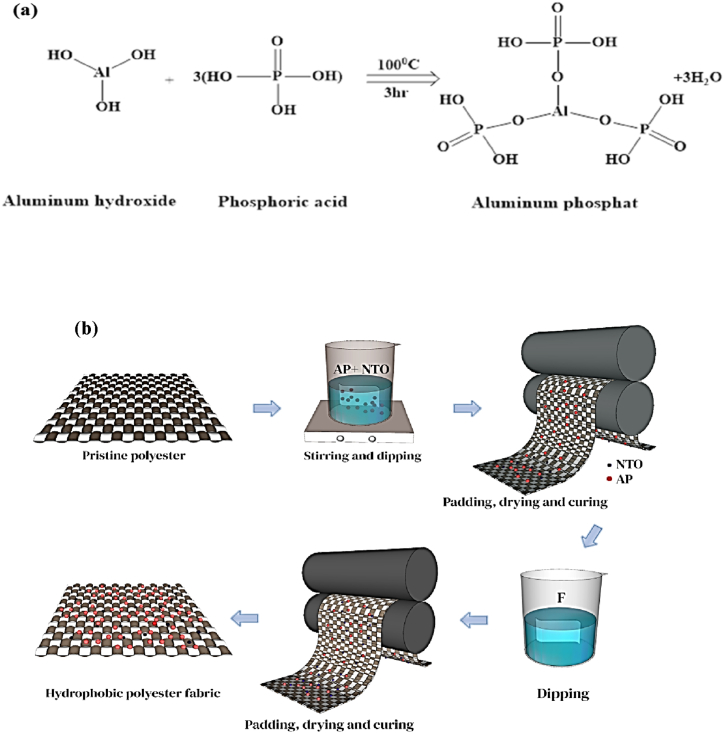
(a) Aluminum phosphate synthesis process by mixing diluted H₃PO₄ (60 %) with Al(OH)₃ in a molar ratio of 3:1 under stirring at 100 °C for 3 h. (b) Two-step procedure for fabricating hydrophobic fabric with the application of TiO₂ nanoparticles coated with aluminum phosphate and fluoropolyacrylate.

AP adhesive was created by blending 9.8 g of diluted H_3_PO_4_ (60 %) with 2.6 g of Al(OH)_3_.in a molar ratio of 3:1 under stirring at 100 °C for 3h (Fig. 1(a)) [31]. On the other hand, NTO particles were dispersed into 10 mL of anhydrous ethanol. After evenly mixing and stirring the above-mentioned suspension was ultrasonicated for 20 min. Subsequently, the polyester fabric was immersed in the coating suspension for 20 s and heated at 180 °C for 5 min named AP-NTO. Eventually, the AP-NTO immersed polyester fabrics were immersed with 2 g L^−1^ fluoroacrylate water-repellent solution and cured at 200 °C for 3 min to obtain more stabilization of NTO particles and reduction of surface energy named as AP-NTO-F (Fig. 1(b)).

### 2.4 Fabrication of Janus fabric featuring a gradient in wettability

A Janus fabric was created by placing the superhydrophobic AP-NTO-F, one side was covered by a glass tape mask, under O_2_/Ar gas with a flow rate of 50 cm^3^ min^−1^ for each gas at 200 W for different specified times (1, 3, and 9 min). The exposed plasma side of the fabric became superhydrophilic, while the other side remained superhydrophobic.

### 2.5 Characterization

The WCA of the fabrics were quantified using a self-made video-optical system with 5 μL of deionized water droplets under normal environmental circumstances. The average WCA value was determined by testing the sample at five distinct points.

The properties of dynamic liquid transport on fabrics were assessed using a moisture management tester (MMT, RF4008MST, REFOND Co., China).

The surface morphology was examined using a field emission scanning electron microscope (FESEM, QUANTA FEG-450, United States) that was equipped with mapping analysis capabilities. The distribution and percentage of elements in the samples were also determined by electron bombardment with energy dispersive X-ray (EDX) analysis.

To assess the fog collection efficiency of the samples, a test setup was built in the laboratory. The sample, measuring 3 cm × 3 cm, was attached to a holder on a glass container. A commercial humidifier with a fog flow with a velocity of approximately of 15 cm s^−1^ was placed at a distance of 10 cm from the sample. The water droplets obtained from the sample are transferred into the container below, and the weight of the container is measured for a period of 5 h. The fog collection efficiency (η) can be determined using Equation (1):

(1) η=MAt

where M (mg) is the mass of water collection, A (cm^2^) is the surface area of the sample, and t (h) is the fog collection time.

The flame retardancy of untreated and treated polyester fabrics was determined using a vertical burning test and the LOI. A vertical burning test was accomplished using a vertical flame tester (CZF-2, Jiangning Analysis Instrument Co., China) in accordance with the ASTM D6413 standard. The LOI value was determined following the guidelines of the ASTM D2863 standard using a limited oxygen indexer (JF-5, Dongguan Yaoke Instrument Equipment Co., China).

The tensile strength of the samples was evaluated using an Instron instrument, which had a gauge length of 20 cm and an expansion rate of 50 mm min^−1^. Three repeated tests were conducted, and the average value was recorded. The air permeability of both the untreated and treated fabrics was measured using the SDL-Air Permeability equipment (TESTEX, Guangdong, China), following the ASTM-D737-75 standard.

The CIE whiteness from wavelengths 360–700 nm was obtained on a reflectance spectrophotometer (Color-Eye 7000A, X-rite, USA) under a standard illuminant/observer D65/10°. Data was gathered at three separate locations for each sample. Subsequently, the mean whiteness degree was determined.

## 3 Results and discussion

### 3.1 Wettability of Janus polyester fabric

Fabrics with spontaneous one-way water transport capability have shown great potential in many fields, including applied textiles, separating water from oil, and collecting fog [32]. Here it is proven that the hydrophobic fabric has the property of one-way water transfer after one side exposure to plasma treatment. Employing a blend of O_2_ and Ar gas to enhance the concentration of oxygen radicals in the plasma helps to increase surface active groups [35]. Unilateral plasma treatment is used to produce unidirectional water transfer fabrics by creating a hydrophobic to hydrophilic gradient through the thickness of the fabric [4]. Fluorocarbon-based hydrophobic compounds revert to their original orientation when exposed to the environment; the NTO particles inhibit this orientation reversion. The NTO increases the surface area available for interaction, enhances the collision of radicals generated by the plasma with the surface, and subsequently improves the presence of surface active groups and hydrophilicity.

**Fig. 2:**
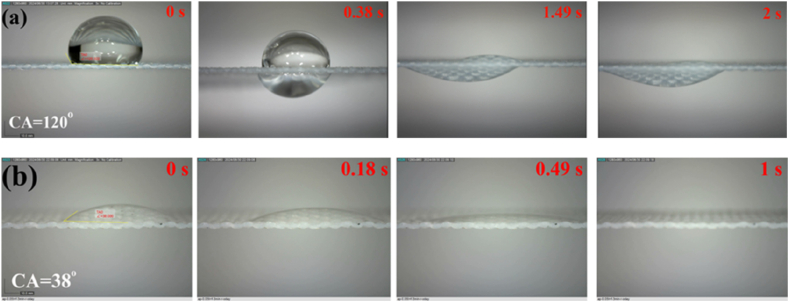
Snapshot of water transfer from two sides of Janus polyester (a) Water drops onto the hydrophobic side and penetrates the fabric spontaneously within 2 s (b) Water drops onto the hydrophilic side and spreads within 1 s.

The water contact angle of the pristine polyester fabric was 0°. When a drop of water was dropped on the untreated polyester fabric surface, it was spread on the surface. The drop was spread in approximately 3 s. Fig. 2(a and b) illustrates the drop of water on either side of the Janus fabric with an asymmetric wettability profile. On the unexposed side, water drops spontaneously penetrated from the hydrophobic side to the hydrophilic side. The duration of this transfer was around 2 s. On the contrary, the drop spreads on the hydrophilic side of the surface without one-way transport of water to the other side. The spreading on the hydrophilic side occurred within around 1 s. Consequently, the Janus polyester fabric achieves diode-like capabilities for water transport, demonstrating considerable one-way transport capability.

Table 2 displays the water transport behavior of fabrics at different times of plasma exposure. Changing the plasma exposure duration altered the hydrophilicity level of the surface, thereby affecting the water transfer characteristics of the fabric. The MMT device can measure the one-way transport capacity (R) parameter, which is the moisture differential between the two surfaces of the fabric [36]. In Zeng et al. investigation, a significant difference in the R value (hydrophobic and hydrophilic side 850 and −157) between the two surfaces of the fabric indicates the movement of water from the hydrophobic side to the hydrophilic side as characteristic of one-way water transport [37]. Therefore, the fabric was hydrophobic on both sides with a plasma time of 1 min (WCA unexposed and exposed side 140° and 135°), and no directional water transfer occurred on the fabric. By extending the plasma exposure to 3 min, the wetting properties of the fabric surface were altered on both sides. The WCA of the plasma exposed side was 35°, while the non-exposed side was 120°. The Janus polyester fabric demonstrated a notable unidirectional transport capability, with R value exceeding 1572 on the unexposed side and R value of −221 on the exposed side. However, when the plasma exposure period was 9 min, the fabric was superhydrophilic on both sides and showed the characteristic of two-way water transport (R unexposed and exposed sides 771 and 732). With the increase in plasma time, the depth of surface modification and oxidation increases; therefore, water transfer changes from one-way to two-way.

**Table 2:** The impact of the duration of O_2_/Ar plasma exposure on the behavior of water transfer in fabric.

Plasma time(min)	WCA(°)		R value		
	Unexposed side to plasma	Exposed side to plasma	Unexposed side to Plasma	Exposed side to Plasma	Water transport
Pristine polyester	0	0	417	411	Two-way
1	140	135	−345	−643	None
3	120	38	1572	−221	One-way
9	25	0	771	732	Two-way

### 3.2 Morphology

**Fig. 3:**
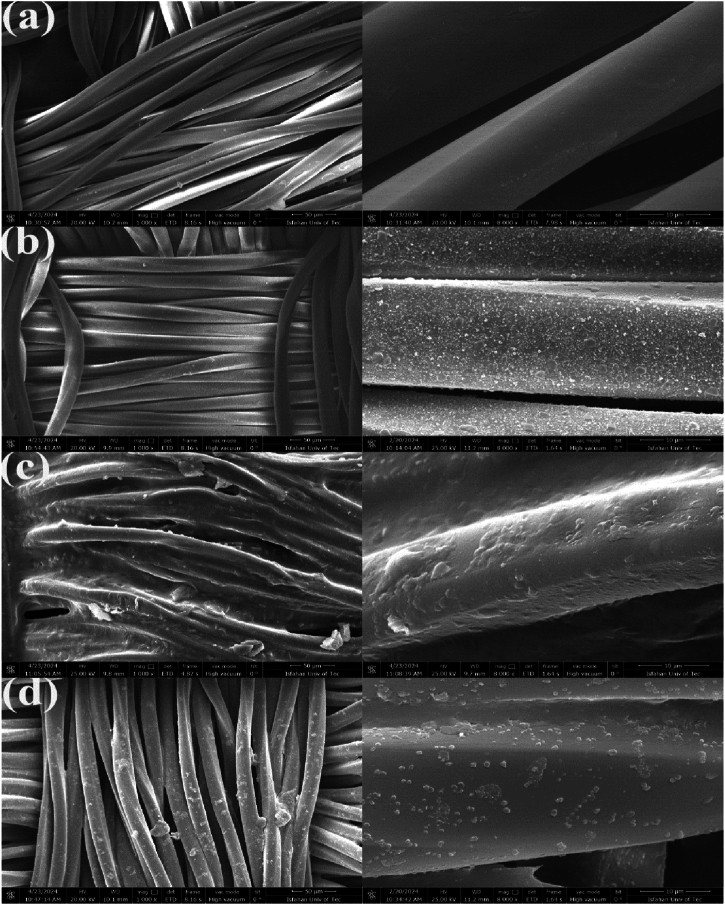
SEM morphologies of (a) smooth surface of untreated polyester fabric, (b) uniform distribution of nanoparticles on the NTO-F@3 sample, (c) strong adhesion of AP on AP-F@3 sample, and (d) attachment of nanoparticles to the surface by AP on the AP-NTO-F@3 sample.

The morphologies of the fabrics after different treatments were determined using scanning electron microscopy (SEM). Imaging was done at two different magnifications for a more detailed comparison of the samples. SEM imaging in Fig. 3(a) indicated that the fibers of the pristine polyester had a clean and smooth surface. Fig. 3(b) illustrates the uniform distribution of NTO particles across the surface of the fiber. As seen in Fig. 3(c), the application of AP mineral adhesive without NTO particles has resulted in the formation of a resinous coating on the surface and the intense adhesion of the fibers to one another. When AP is used in the presence of NTO particles, it attaches the NTO particles to the surface of the fabric. Fig. 3(d) clearly shows how the AP coated the NTO particles, leading to the formation of a hierarchical roughness structure on the fabrics at the micro-nano scale. It can also be seen that the plasma treatment has preserved the structure of the fibers without any breakage.

**Fig. 4:**
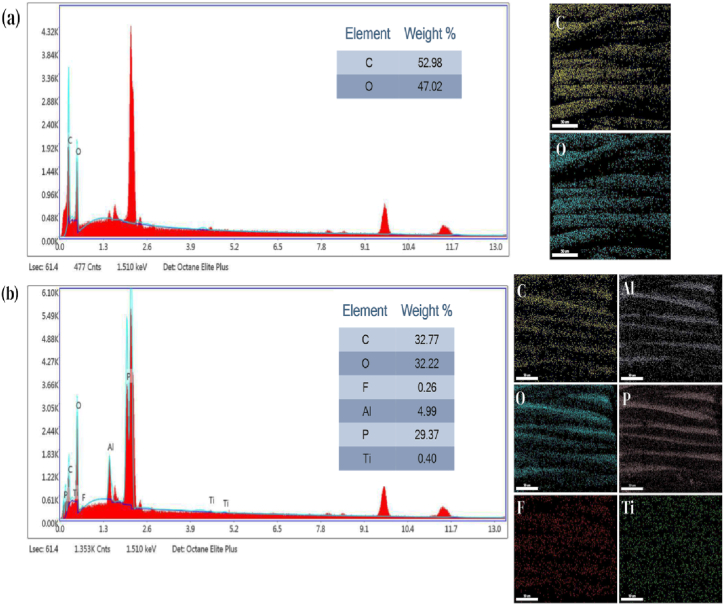
(a) Element mapping and the EDS spectrum of C and O of untreated fabric surface, and (b) Element mapping of C, O, F, P, Al and Ti of EDS spectrum from AP-NTO-F@3.

The surface elemental composition and distribution of the original fabric and the AP-NTO-F@3 Janus fabric were analyzed using the EDS spectrum. The surface of the original fabric is composed mostly of two elements: carbon (C) and oxygen (O). The content of C is 52.98 %, and the content of O is 47.02 % (Fig. 4(a)). The Janus fabric surface mostly consists of carbon (C), oxygen (O), aluminum (Al), phosphorus (P), titanium (Ti), and fluorine (F), as demonstrated in Fig. 4(b). The EDS mapping images indicated a uniform distribution of these elements on the polyester fabric. The presence of P and Al is ascribed to the AP adhesive, while F denotes fluoroacrylate polymer. The oxygen content is 32.22 %, and the carbon content is 32.77 %.

### 3.3 Efficiency of fog collection

**Fig. 5:**
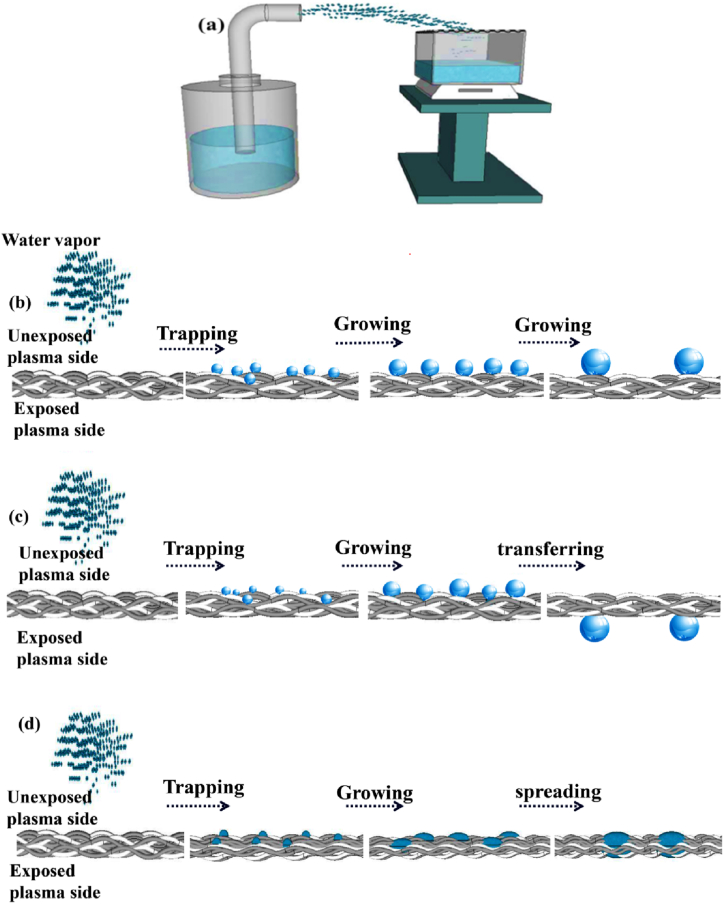
(a)Schematic of the self-made fog-collecting system. Effects of plasma exposure on fog collection for three different durations: (b) 1, (c) 3, and (d) 9 min. Water droplets on the fabric exposed to plasma for 1 min increased in size and adhered to the surface, for 3 min, contacted water droplets grew and vertically transported to the superhydrophilic surface and for 9 min, contacted water droplets grew and spread vertically and horizontally.

**Fig. 6:**
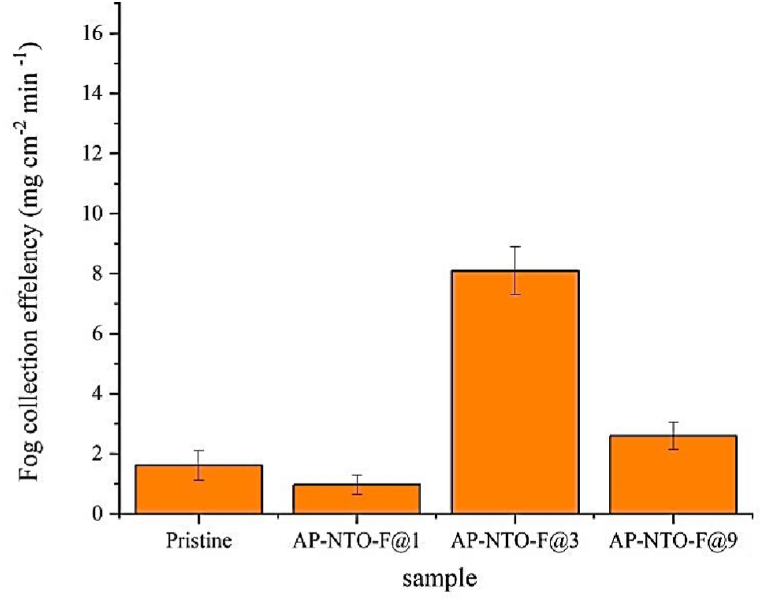
Fog collection efficiency for various samples.

An analysis was conducted of the varying behaviors of fog at distinct plasma periods to understand the mechanism of the improved fog collection. Fig. 5(a) depicts a fog collection system where the surface with hydrophobic properties is oriented toward the fog droplets, and the surface with hydrophilic properties is oriented toward the glass container. Additional information is provided in the experimental section. According to Fig. 5(b) and the Cassie model, when the fog made contact with the surface exposed to plasma for 1 min the water on the fabric surface only made contact with the upper side of the rough area, while air pockets were trapped beneath the liquid, creating a state where the fabric did not become wet. Ultimately, due to water blockage, the water did not transfer to the container and had the least effect on fog collection, leading to the lowest effectiveness in collecting fog. Fig. 5(c) demonstrates the fog collection behaviors of the Janus fabric at a plasma time of 3 min. The water droplets stuck to the fabric surface and grew in size as more fog passed through. The droplets of water were rapidly drawn downward due to the capillary force exerted by the underlying superhydrophilic surface and collected under gravity in the bottom container. The hydrophobic surface remained dry. The transportation of all water droplets to the bottom container resulted in a very efficient ability to collect fog. The Janus fabric had a fog collection efficiency of around 8.1 mg cm^−2^ min^−1^(Fig. 6), demonstrating markedly superior performance compared to those research-related polyester fabrics (Table 1). The utilization of mesh structures faces the challenge of mesh design. The fine mesh is inefficient in capturing fog droplets, whereas the mesh with a high mesh number is susceptible to surface blockage during fog collection. Here, by altering the plasma duration, a surface leads to the droplets nucleation and transfer through the fabric. When exposed to plasma for 9 min, water droplets increased in size as they made contact with the fabric surface and then quickly spread due to the capillary force resulting from its superhydrophilicity (Fig. 5(d)). This fabric exhibited a fog collection efficiency of approximately 2.6 mg cm^−2^ min^−1^ (Fig. 6).

### 3.4 Theoretical framework for the process of one-way water transfer

The interface between water and solid was examined to understand the transfer mechanism. The roughness of micro-nano structures with the fluorocarbon hydrophobic material creates a hydrophobic force on the surface [38,39]. According to the petal phenomenon, a drop of water adheres to the fabric surface in a perfectly spherical shape exhibiting strong resistance to movement even when the surface is turned upside down [40]. With this phenomenon, an explanation can be provided for this mechanism. Incursion pressure is an important parameter in the drop-transfer process. This pressure must be large enough for a transfer to occur. The intrusion pressure, ΔP, can be defined as follows:

(2) $$\Delta P=\frac{2\gamma \;\cos\;\theta }{R}$$

where γ is the interfacial tension; θ is the advancing water contact angle, and R is the radius of pore [41].

**Fig. 7:**
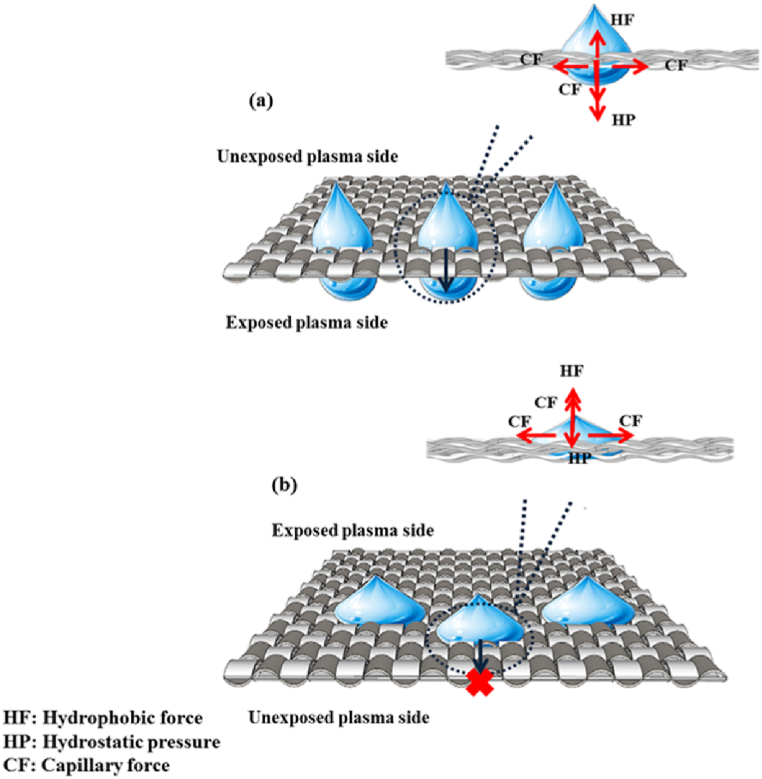
Schematic representation of one-way water transport mechanism (a) The droplet moves from the hydrophobic side to the hydrophilic side (b)The movement of the droplet from the hydrophilic side to the hydrophobic side.

As seen in Fig. 7, when water contacts the superhydrophobic surface, the hydrostatic pressure causes the water to permeate the fabric. However, the hydrophobic force acts as an opposing force, preventing penetration. Equation (2) assumes that the pore radius and surface tension remain consistent on both sides of the fabric, whereas only the contact angle varies. It is obvious that if θ is greater than 90°, ΔP > 0. The hydrostatic pressure is large enough to overcome the hydrophobic force, and the water drop penetrates the fabric. Once the water reaches the hydrophilic side of the fabric, the capillary force, along with the hydrostatic pressure, helps the water penetrate the fabric. Thus, supplying water from the hydrophobic side possibly leads to water transport to the opposite side (Fig. 7(a)). While water supply from the hydrophilic side causes the water to spread across the surface due to capillary force. Therefore, θ is less than 90° and ΔP < 0. Consequently, the hydrostatic pressure is insufficient to overcome the hydrophobic force (Fig. 7(b)).

### 3.5 Flame retardant properties of Janus polyester fabrics

**Fig. 8:**
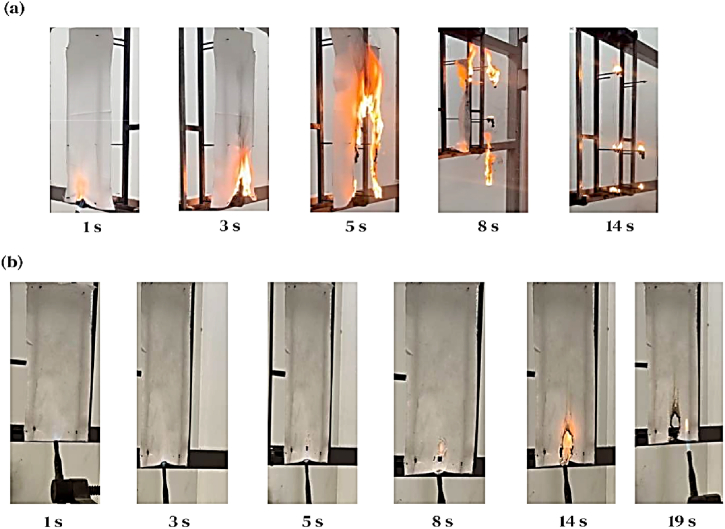
Digital images of polyester fabrics after vertical burning test (a) High flammability of pristine and (b) Exceptional flame resistance of AP-NTO-F@3 fabric.

To increase safety and reduce the risk of fire, flame-resistant fabrics were considered. Table 3 records the measured data, including after-flame time, char length, and LOI value, to investigate the flammability behavior of untreated and different treated samples. Fig. 8(a) illustrates the high flammability of untreated fabric as the flame spread to the end of the polyester for 8 s. The after-flame time, char length, and LOI were 16 s, 300 mm, and 20 %, respectively. Additionally, heavy dripping occurred during the burning process. However, following treatment with AP-NTO-F@3, the fabric exhibited exceptional flame resistance and anti-dripping properties due to the intrinsic thermal resistance of AP (Fig. 8(b)) [25]. Further, the fabric was immediately extinguished upon the removal of the flame. The LOI value was measured at 32.8 %, indicating the lowest amount of oxygen required to sustain combustion. The length of the char was reduced to 70 mm. Inorganic compounds such as (NTO/AP) are non-flammable owing to their high thermal resistance. They function as ash, inhibiting the transmission of heat and oxygen. Given the minimum NTO utilized to decrease the plasma duration in the method applied in this experiment, the fabric treated with AP-F@3 exhibited flame-retardant properties similar to the fabric treated with AP-NTO-F@3.

**Table 3:** Flame retardant and dripping properties of pristine and different treated samples.

Samples	After-flame time (s)	Char length (mm)	LOI (%)	Dripping
Pristine polyester	16	300	20	Heavy
AP-F@3	0	72	31.4	No
AP-NTO-F@3	0	70	32.8	No

### 3.6 Mechanical and physical characteristics

**Fig. 9:**
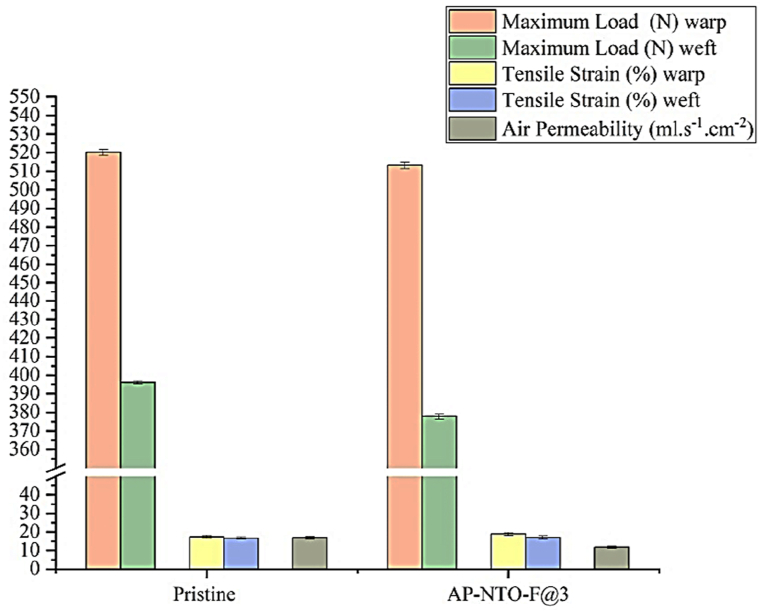
Mechanical and physical characteristics of the pristine and Janus fabrics.

**Fig. 10:**
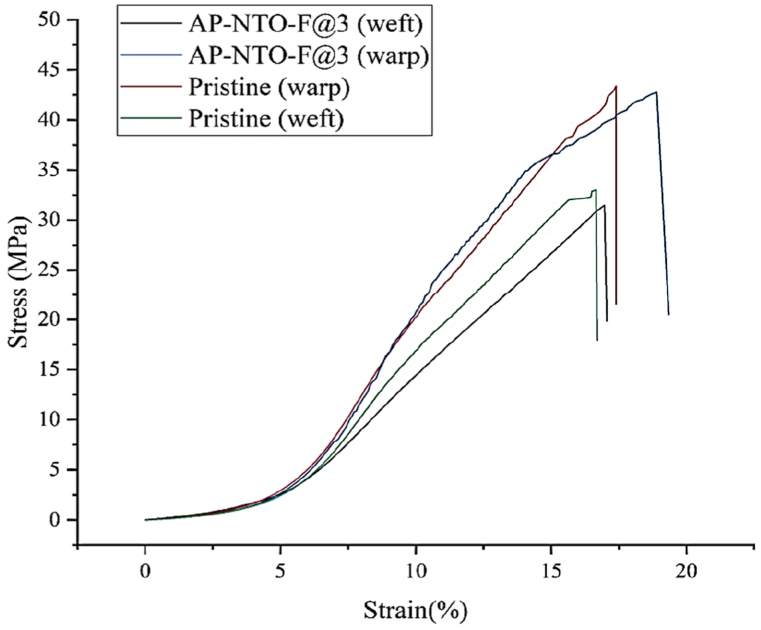
Stress-strain of the pristine and AP-NTO-F@3 polyester fabrics.

The mechanical and physical characteristics of the untreated and treated fabrics were compared based on the tensile strength and air permeability data presented in Fig. 9. The AP-NTO-F@3 fabric shows no signs of corrosion or cracks on its surface (Fig. 3(d)). Hence, there are no significant changes in the maximum force (513.2 N) compared to the untreated fabric (520.3 N). Also, the stress-strain curves exhibited no significant change in the force required to tear the fabric in both the warp and the weft directions (Fig. 10). The air permeability of the Janus polyester fabrics was inspected by measuring the rate of air flow passing through a certain surface area of the fabric at 20 Pa. Fig. 9 demonstrates that the air permeability of the AP-NTO-F@3 fabric is reduced when compared to the untreated polyester fabric. This could be due to the AP coating on spaces between the fibers within the fabric structure.

### 3.7 Whiteness index

The degree of whiteness of the samples was measured using a reflective spectrophotometer and whiteness. The results obtained are displayed in Table 4. The samples treated with AP-F@3 showed the most significant changes in whiteness. AP reduces the degree of whiteness. Hornsveld et al. indicated that the refractive index of aluminum phosphate, with a P:Al ratio of 3:1 at 180 °C, is below 1.47, which is inferior to the refractive index of polyester, thereby reducing the whiteness of the polyester fabric [42]. Since NTO particles have a low absorption coefficient in the visible range and a high refractive index (n = 2.7 and 2.45 for rutile and anatase phases, respectively), it reduced the changes in the whiteness of the samples [43]. Consequently, NTO besides reducing plasma duration can partially mitigate the reduction in whiteness. The color with more light absorption generates higher energy. However, whether energy can increase the transmission or evaporation of droplets requires further investigation. Here, the color changes are very low that can be ignored.

**Table 4:** Whiteness index of pristine and different treated samples.

Samples	WI (CIE)
Pristine polyester	71.6
F@3	71.3
AP-F@3	48.8
AP-NTO-F@3	59.5

## 4 Conclusions

In summary, the Janus polyester fabric was produced with several features, including one-way water transfer, fog collection, mechanical durability, and flame resistance. This was achieved by applying a hydrophobic coating of NTO particles, AP, and F on the fabric, followed by treatment with one-sided O_2_/Ar plasma. The surface roughness generated by AP and NTO, in combination with the hydrophobic fluorocarbon material, resulted in the creation of a hydrophobic force on the surface. Plasma enables one-way water transfer through the fabric by establishing a wetting gradient in the hydrophobic substrate. The fabric exhibits one-way water transport characteristics only when the duration of plasma exposure is within a specific range. When the fabric was exposed to plasma for 3 min, it showed the property of unidirectional water transport (R = 1572 %). Among all fabrics, this fabric exhibited the highest fog collection efficiency, measuring 8.1 mg cm^−2^ min^−1^. With the increase in plasma time due to the increase in the depth of modification and oxidation of the surface, the transfer of water changed from one-way to two-way. Due to the high thermal resistance of AP, the fabric showed exceptional flame resistance and anti-drip properties. This finishing operation did not affect the tensile strength of the fabric.

## CRediT authorship contribution statement

**Najmeh Izadi:** Writing – original draft, Visualization, Investigation, Data curation. **Majid Montazer:** Writing – review & editing, Visualization, Validation, Supervision, Resources, Project administration, Funding acquisition, Formal analysis, Conceptualization. **Aminoddin Haji:** Supervision, Resources.

## Data availability statement

Data will be made available on request.

## Declaration of competing interest

The authors declare that they have no known competing financial interests or personal relationships that could have appeared to influence the work reported in this paper.
